# Toward a Multivalent Synthetic Oligosaccharide-Based Conjugate Vaccine against *Shigella*: State-of-the-Art for a Monovalent Prototype and Challenges

**DOI:** 10.3390/vaccines10030403

**Published:** 2022-03-07

**Authors:** Armelle Phalipon, Laurence A. Mulard

**Affiliations:** 1 Institut Pasteur, Innovation Lab. Vaccines, F-75015 Paris, France; 2 Institut Pasteur, Université de Paris, CNRS UMR3523, Unité Chimie des Biomolécules, F-75015 Paris, France

**Keywords:** carbohydrates, conjugate vaccines, glycoconjugates, O-antigens, *Shigella*, synthetic glycans

## Abstract

This review focuses on the molecular glycovaccine concept, a promising option to develop a *Shigella* glycoconjugate vaccine. Subsequent to original developments involving, as main vaccine component, the detoxified *Shigella* lipopolysaccharide randomly conjugated at multiple sites to a carrier protein, novelty stems from the use of rationally designed, well-defined chemically synthesized oligosaccharide haptens conceived as functional surrogates of the main surface antigen, linked via single-point attachment onto a carrier. The concept and design of such a fine-tuned *Shigella* glycovaccine are presented by way of SF2a-TT15, a neoglycoprotein featuring a synthetic 15-mer oligosaccharide, which constitutes an original vaccine prototype targeting *Shigella flexneri* 2a, one of the predominant circulating strains in endemic settings. The clinical testing of SF2a-TT15 is summarized with the first-in-human phase I trial in young healthy adults showing a good safety profile and tolerability, while inducing bactericidal antibodies towards *S. flexneri* 2a bacteria. The proof-of-concept of this novel approach being established, an ongoing phase IIa clinical study in the nine-month-old infant target population in endemic area was launched, which is also outlined. Lastly, some challenges to move forward this original approach toward a multivalent cost-effective *Shigella* synthetic glycan conjugate vaccine are introduced.

## 1. Introduction

Diverse strategies have been pursued to develop a *Shigella* vaccine over the past 100 years. Only two of them have reached phase III clinical trials, namely, orally administered live attenuated strains and parenterally administered polysaccharide-protein conjugates. Stemming from their previous achievements in the field of *Haemophilus influenzae* b (Hib) vaccination, the concept of *Shigella* polysaccharide-protein conjugate vaccines was originally introduced by John B. Robbins and colleagues [1]. Briefly, bacterial polysaccharides that are key targets of the naturally induced immunity are well-known T-cell independent antigens. Their conjugation to a carrier protein enables the induction of the desired T-cell dependent humoral immunity, including priming of the host memory B cells (for a review see [2]). Of note, several multivalent conjugate vaccines have been successfully implemented against diseases caused by capsulated bacteria, the highest strain coverage being achieved so far with the 13-valent *Streptococcus pneumoniae* licensed vaccine (for a review see [2]).

For *Shigella*, the main target of protective antibodies is the capsular polysaccharide when present or the O-antigen (O-Ag) part of the lipopolysaccharide (LPS) [3]. Having hypothesized in the early 1990s that serum antibodies to *Shigella* O-Ag could provide protection by transudation to the intestinal mucosal surface and bacteria inactivation in the intestine [1,4], the Robbins’ and Schneerson’s group at the National Institutes of Health (NIH) extensively investigated the use of detoxified LPSs as the basis for parenteral *Shigella* glycoconjugate vaccines. A diversity of “lattice”-type *Shigella* conjugates—abbreviated as NIH conjugates—in which the detoxified LPS and the carrier protein are covalently linked at multiple sites, were generated. The proof of concept of their safety, immunogenicity and protective efficacy was established in young adults and children (for a review, see [5]). However, the lack of efficacy of the most advanced *S. sonnei* NIH vaccine prototype in the main target population of *Shigella* infection, i.e., one-to-two-year-old children, encouraged the search for alternatives to this first generation of conjugate vaccines [5]. Going from concept to phase II clinical trial, the following provides an overview of our achievements in the field of synthetic glycan-based *Shigella* vaccines with focus on SF2a-TT15, a “sun”-type synthetic glycan-tetanus toxoid (TT) conjugate conceived as a promising *S. flexneri* 2a (SF2a) vaccine candidate. Diverging from other options under investigation, the concept of synthetic carbohydrate hapten takes advantage of the versatility of chemical synthesis and its potential when aiming at immunogens fine-tuned to drive the antibody response towards the key protective determinants of the native surface polysaccharide. Providing support to developments ongoing at Institut Pasteur was a report in 1999 by the NIH team on the superior immunogenicity of synthetic oligosaccharide-HSA (Human Serum Albumin) “sun”-type conjugates compared to a “lattice”-type counterpart targeting *S. dysenteriae* 1 [6].

## 2. From Polysaccharide Antigens to SF2a-TT15, a *Shigella* Synthetic Glycan Conjugate Vaccine Prototype

### 2.1. Concept: Synthetic Glycans as Surrogates for Shigella O-Ags

**Figure 1:**
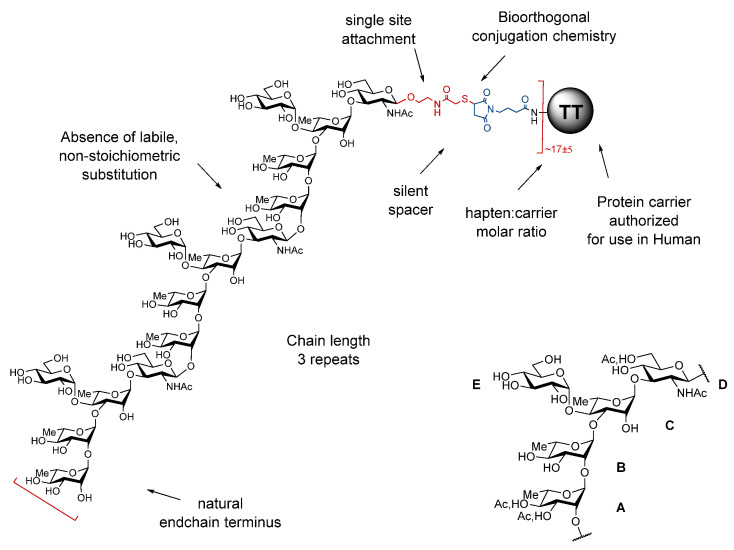
Key parameters governing the design of “sun”-type synthetic glycan-based conjugates exemplified for SF2a-TT15. Top left: SF2a-TT15. Bottom right: Biological repeating unit of the SF2a O-Ag [7]. A = B = C: l-rhamnopyranose, D: *N*-acetyl-d-glucosamine, E: d-glucopyranose, Ac: acetyl, TT: tetanus toxoid.

Bacterial O-Ags are defined by linear or branched repeats made of up to eight monosaccharide residues. They feature tremendous disparities in terms of chain length and often owing to the presence of non-stoichiometric labile and/or phase-associated substitutions, which may be essential components of the protective epitopes. By essence, detoxified LPSs are therefore highly heterogenous molecules. Moreover, despite major improvements over the past decades, conjugate manufacturing is not without risk. The chemical manipulations necessary for LPS extraction, detoxification, and subsequent conjugation of the polysaccharide material to a suitable carrier contribute to deliver complex poorly defined glycoconjugates, especially when involving random conjugation at multiple sites on the polysaccharide component. Key epitopes may be altered and labile O-Ag substitutions may not survive the process whilst neo-epitopes may be generated. As a result, partial loss of immunogenicity is not unexpected and quality control is a highly demanding process. In contrast, the use of a well-defined synthetic O-Ag surrogate, preferably a fine-tuned oligosaccharide, equipped with a unique orthogonal reactive moiety provides neoglycoproteins, whereby a suitable carrier protein is covalently attached to multiple versions of the selected hapten under a controlled manner. The foundation of the strategy resides in the early acknowledgement that despite bacterial polysaccharides being large polymers, anti-polysaccharide monoclonal antibodies recognize glycotopes made of up to six-eight residues and that sera induced in mice by a disaccharide-protein conjugate could confer resistance to a challenge with *S. pneumoniae* type 3 bacteria in rabbit. The obtained “sun”-type glycoconjugates are best defined in terms of protein, oligosaccharide and linker components as well as conjugation chemistry, site of covalent attachment, and oligosaccharide:protein molar ratio, respectively (Figure 1). Available data suggest that these different parameters are inter-related and that they all contribute to the immunogenic properties of the obtained glycoconjugates [5].

As implemented, the identification of the oligosaccharide component was best achieved according to a stepwise strategy synergizing between medicinal chemistry, structural vaccinology and immunochemistry. Of note, knowledge of the exact nature of the O-Ag repeating unit is an absolute pre-requirement that governs hapten selection according to this multidisciplinary strategy. Besides chain length, which translates into the number of basic O-Ag repeating units, the nature of the endchain residue and the possible presence of non-carbohydrate substitutions are essential features to take into consideration (Figure 1).

### 2.2. Design and Properties of SF2a-TT15, a “Sun”-Type Synthetic Oligosaccharide-Based Conjugate

The SF2a O-Ag is defined by a branched pentasaccharide basic repeating unit, depicted as AB(E)CD (Figure 1), partially O-acetylated at two sites [7]. At the time the NIH conjugates were developed, the O-acetylation pattern and the detailed structural elements composing the protective epitopes were unknown. Taking advantage of TT as a well-established carrier for human vaccines, focus was on the identification of a suitable synthetic O-Ag surrogate. This was achieved by use of five murine protective monoclonal IgG antibodies (mIgGs) and a large panel of synthetic O-Ag substructures, up to a 20-mer oligosaccharide i.e., a four-repeat O-Ag segment. A two-step process was engaged whereby oligosaccharides pre-selected in vitro as potential antigenic O-Ag mimics were turned into glycoconjugates for in vivo assays and fine selection as functional mimics, i.e., for their ability to induce high titers of protective anti-SF2a LPS IgG antibodies in mice [5]. Briefly, important conclusions were as follows:

- While the branched B(E)CD segment was identified as a minimal antigenic determinant, the B(E)CD-TT conjugate did not induce any anti-SF2a LPS antibodies in mice despite eliciting high anti-B(E)CD IgG antibody titers. This discrepancy between antigenic mimicry and functional mimicry provided a strong support to a deeper molecular investigation on larger O-Ag segments, also taking into account conformational and structural mimicry.
- Antibody binding increased with chain length to reach a plateau for oligosaccharides larger than B(E)CDAB(E)C, suggesting that antigenic mimicry required oligosaccharides longer than one repeat. This observation was comforted by the determination of the crystal structure of a mIgG in complex with a synthetic 15-mer ([AB(E)CD]_3_) segment. The antibody binding site accommodates a 9-mer glycotope. Six residues located on two adjacent repeats make direct contact with the antibody, suggesting that a suitable O-Ag surrogate should comprise at least two contiguous repeats to achieve structural O-Ag mimicry.
- NMR data in solution revealed strong signal overlap for internal residues only within the 10-mer ([AB(E)CD]_2_) and 15-mer ([AB(E)CD]_3_) segment, suggesting that O-Ag conformational mimicry was best reached for the latter.
- Recognition of [AB(E)CD]_3_ by sera from naturally infected or vaccinated individuals, was superior to that of [AB(E)CD]_2_ and paralleled that of the LPS isolated from the SF2a strain used to generate mIgGs.
- Moreover and comforting the above findings, binding data for all five mIgGs revealed that O-acetylation was not a critical feature of SF2a protective epitopes.
- The blockwise synthesis established at the lab scale of the ready-for-conjugation 15-mer oligosaccharide equipped with an aminolinker at its reducing end [8] was not considered more demanding than that of the 10-mer equivalent, suggesting that chemical synthesis was not a limiting factor.
- The immunogenicity of a non-adjuvanted [AB(E)CD]_3_-TT conjugate far exceeded that of a [AB(E)CD]_2_-TT conjugate featuring a similar average oligosaccharide:TT molar ratio, while no detrimental anti-linker antibody titers were detected. In addition, conjugates encompassing larger O-Ag synthetic segments or oligosaccharides differing by their endchain residue did not surpass the 15-mer conjugate (Mulard et al., unpublished data).

On this basis, the synthetic 15-mer [AB(E)CD]_3_, representing three basic O-Ag biological repeats, was selected as a promising antigenic, structural, conformational, and functional mimic of the natural antigen. Advantageously for manufacturing purpose, [AB(E)CD]_3_ is not O-acetylated, suggesting an enhanced chemical stability and easier quality controls than for the natural O-Ag, as underlined for classical polysaccharide conjugate vaccines [9]. Taking advantage of the high-yielding thiol-maleimide conjugation chemistry, an optimal glycoconjugate composition, named SF2a-TT15 (Figure 1), was identified in the form of an oligosaccharide:protein molar ratio of 17 (± 5) giving rise, when formulated with aluminum hydroxide (AlH), to long-lasting protective SF2a LPS-specific IgGs in mice [10]. The original synthesis of SF2a-TT15 was scaled up successfully. A GMP batch was produced [11]. SF2a-TT15^GMP^ was immunogenic in rabbits and passed all toxicity criteria [11]. Moreover, SF2a-TT15-induced sera in mice recognized a large panel of SF2a strains circulating worldwide [11]. Based on these data, this original vaccine prototype was move forward into a first-in-human evaluation.

## 3. SF2a-TT15: Safety, Tolerability, and Immunogenicity in a First-in-Human Phase I Study

A dose-escalating, single-blind, observer-masked, randomized, placebo-controlled study was performed at the Tel Aviv Sourasky Medical Center in Israel (ClinicalStudies.gov, NCT02797236) [12]. A summary of the study design as well as key safety and immunological outcomes are presented below.

Two cohorts of 32 participants each were settled to receive 2 µg (cohort 1) and 10 µg (cohort 2) carbohydrate dose of SF2a-TT15 non-adjuvanted or adjuvanted with AlH or matching placebos, administered as three intramuscular injections, 28 days apart. Thus, in each cohort, 12 participants received the adjuvanted vaccine and 12 received the non-adjuvanted formulation, while two groups of four participants each received the matching placebos. The tolerability and safety profile were good at both doses with no occurrence of serious or severe adverse events. This is consistent with the general good safety record of polysaccharide-protein conjugate vaccines and specifically with that of the NIH SF2a-rEPA conjugates [5] and more recently Flexyn2a, a bioconjugate vaccine candidate [13].

All the immunological outcomes evaluated in this clinical study show that both the low and high oligosaccharide doses of SF2a-TT15, adjuvanted or not, are immunogenic. No variation over time in the baseline for the tested immunological parameters was noticed in placebo recipients, indicating the absence of natural exposure to SF2a LPS or any cross-reacting antigens in the study population during the follow-up. Therefore, the observed immune response results exclusively from the stimulation of the host immune system by the vaccine candidate. Focusing on the anti-SF2a LPS IgG response, IgG2 titers predominantly increase, followed by IgG1 titers, similarly to the pattern elicited by the first generation of SF2a conjugates and natural SF2a infection. Compared with the low dose, the 10 µg carbohydrate dose is much more potent, inducing stronger anti-SF2a LPS IgG titers both in magnitude and percentage of responders, from the first injection with no additional effect of the subsequent ones. The absence of boosting has also been reported following vaccination of Israeli adult volunteers with the NIH SF2a conjugates [5] administered at higher doses, and more recently in American adult recipients of Flexyn2a at a 10 µg polysaccharide dose regimen [13]. Adjuvanting with AlH improved the anti-SF2a LPS IgG titers induced by the low, but not the high SF2a-TT15 dose. It also contributed to the sustained antibody response measured at three months after the third injection for both doses. These results differ from the observation that AlH had no impact on the immune response of adult volunteers, at least 28 days after one or two injections, to the SF2a lattice-type conjugate and bioconjugate vaccines, respectively [5,13]. In the case of SF2a-TT15, both the evaluation of a lower carbohydrate dose and the longer follow-up after the 10 µg dose might have uncovered the added value of the use of AlH as adjuvant. The functionality of vaccine-induced antibodies as measured by serum bactericidal activity (SBA) was robust whatever the saccharide dose used. A strong correlation was reported between SBA and the magnitude of the SF2a-TT15-induced anti-SF2a LPS IgG response. Significant rises in the anti-SF2a LPS IgG memory B-cell percentage was observed for both doses, with the high dose formulations out-performing the low ones, suggesting a successful priming and longevity of the anti-SF2a immune responses induced by this fine-tuned synthetic glycan-based conjugate. While no data are available for the first *Shigella* polysaccharide conjugates, such a memory B-cell priming was recently reported for Flexyn2a [14].

The potential of *Shigella* glycoconjugate vaccines to stimulate the immune system of naive individuals is a matter of debate. In particular, concern arose from the lack of immunogenicity, correlated to the lack of protection, observed in recipients of the NIH *S. sonnei* conjugate under the age of three, whereas older children and young adults, supposedly immunologically primed by natural infection, were protected [15]. In this context, the impact of pre-existing immunity on the immunogenicity of SF2a-TT15 comes into question. However, detectable serum IgG antibodies to SF2a do not necessarily mean pre-exposure to homologous bacteria. They can reflect cross-reactivity with LPSs from other Enterobacteriaceae, shown to increase with age and reported in previous *Shigella* vaccine studies in the USA or Europe [3]. More importantly, the extent of the naive status in the target population, i.e., nine-month-old infants in LMICs, is being questioned. Indeed, accumulating epidemiological data [16] show that there is already substantial exposure to SF2a at three to six months of age. Therefore, conducting a phase IIa study in the target population in endemic settings is the only option to assess the real potential of this unique type of *Shigella* vaccine.

## 4. SF2a-TT15: Ongoing Phase IIa Clinical Study to Assess Safety, Tolerability and Immunogenicity in the Target Infant Population Living in Endemic Areas

Based on the good safety profile and immunogenicity of SF2a-TT15 in young adults in a high-income country setting, together with its suitable stability profile, a single site, double-blind, observer-masked, randomized, placebo-controlled, age-descending phase IIa study was launched at Kenya Medical Research Institute (KEMRI), Kericho, Kenya (ClinicalTrials.gov Identifier: NCT04602975). The purpose of this study is to examine in nine-month-old infants, the safety and immunogenicity of two vaccine doses—2 µg and 10 µg of oligosaccharide equivalent adjuvanted or not with AlH, respectively, as previously tested in the phase I trial [12]—after two intramuscular injections three months apart, followed by a third injection six months later. Of note, at the second and third injections, the infants will be concomitantly administered with SF2a-TT15 and a measles-rubella (MR) vaccine (one per arm) to match with the MR kenyan expanded program of vaccination. Based on safety and tolerability evaluation after the first injection, an independent data safety monitoring committee (IDMC) will give recommendation to move from the adult cohort 1 (18–50-year-old subjects, 12 vaccinees receiving the 10 µg oligosaccharide dose of the vaccine formulated with AlH and four placebos) to the children cohort 2 (two-to-five-year-old subjects, same dosing and number of subjects as cohort 1), and then to the nine-month-old infants cohort 3. For the latter, IDMC recommendation will allow to move from cohort 3A receiving the 2 µg oligosaccharide dose with and without AlH (40 subjects each plus 10 subjects for each matching placebo, thus a total of 100, cohort 3A) to cohort 3B receiving the 10 µg oligosaccharide dose adjuvanted or not, with the same distribution of subject number as for cohort 3A.

The study has started in October 2020 and is progressing well: cohorts 1 and 2 have received the three injections, cohorts 3A and 3B have received the second and first injection, respectively. The final report, including a 6-month follow-up after the third injection, is expected by mid-2023.

Not the least and in complement to the above, a controlled human infection model study (CHIM, phase IIb clinical trial) was approved to assess the protective efficacy of SF2a-TT15 while confirming its tolerability, reactogenicity, and immunogenicity in supposedly *Shigella* non-exposed western volunteers. The study was launched at the Center for Vaccine Development (CVD, University of Maryland, Baltimore, MD, USA) in February 2022 (ClinicalTrials.gov Identifier: NCT04078022).

## 5. Toward a Multivalent Synthetic Glycan-Based *Shigella* Vaccine Providing Broad Strain Coverage

**Figure 2:**
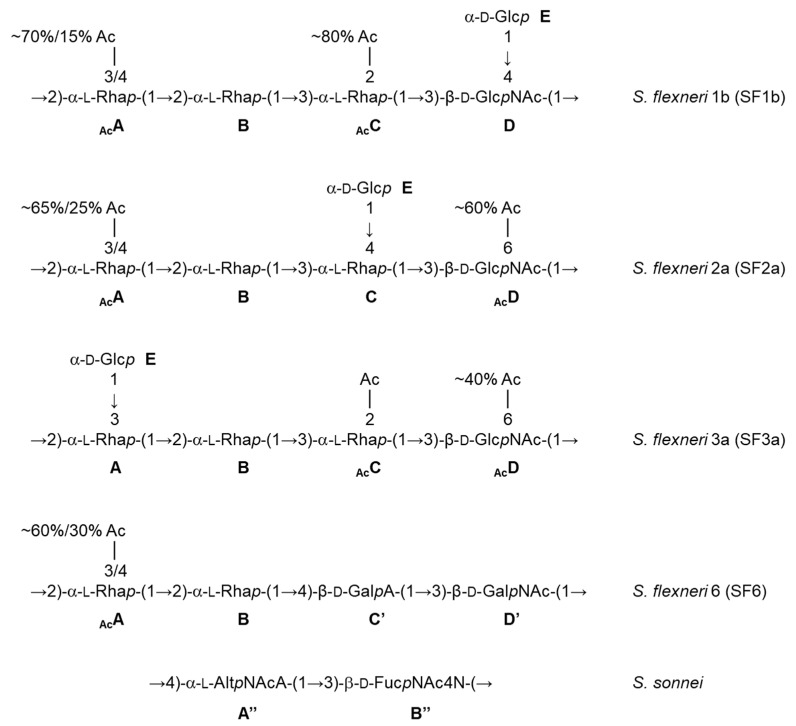
Repeatings units of the *Shigella* O-Ags under consideration for the development of a broad strain coverage *Shigella* vaccine [7,20]. Ac: acetyl, Rha*p* (A,B,C): rhamnopyranose, Glc*p*NAc (D): 2-acetamido-2-deoxy-glucopyranose (*N*-acetyl-glucosamine), Glc*p* (E): glucopyranose, Gal*p*A (C’): galactopyranuronic acid, Gal*p*NAc (D’): 2-acetamido-2-deoxy-galactopyranose (*N*-acetyl-galactosamine), Alt*p*NAcA (A”): 2-acetamido-2-deoxy-altropyranuronic acid (*N*-acetyl-altrosaminuronic acid), Fuc*p*NAc4N (B”): 2-acetamido-4-amino-2,4,6-trideoxy-galactopyranose (AAT).

Besides promoting the phase II clinical trials, the feasibility and successful outcome of the phase I study with the monovalent SF2a-TT15 vaccine prototype revealed the strategy and fostered its further development to provide a multivalent *Shigella* glycovaccine suited to answer the need in the field, i.e., to cover a highly diverse panel of circulating strains. In fact, promising original preclinical data obtained early on for SF2a-TT15 [17] incited upstream investigation to identify O-Ag synthetic oligosaccharide mimics of the predominant *Shigella* circulating strains as updated [18], also considering the potential for cross-reactivity based on O-Ag repeats and polysaccharide dynamics [7,19]. As briefly highlighted below, the very diverse O-Ags under consideration (Figure 2) generated major synthetic challenges, all of which were overcome to achieve convincing synthetic glycan-based immunogens and promising combinations thereof (Mulard et al., unpublished data). Of special interest, the *S. sonnei* O-Ag represents a unique challenge owing to its zwitterionic disaccharide repeat [20]. Nevertheless, oligosaccharides representing O-Ag segments of various composition and length were synthesized [21,22], paving the way to a library of neoglycoconjugates demonstrating remarkable immunogenicity in mice [23]. Alternatively, the synthesis of a large set of *S. flexneri* 6 (SF6) oligosaccharides enabled the identification of an immunodominant epitope central to further developments (Chassagne et al., unpublished data) [24]. Promising immunogenic SF6 conjugates differentiated by their synthetic oligosaccharide component were achieved (Bouchet et al., unpublished data). Likewise, the SF2a experience was exploited for *S. flexneri* 3a (SF3a). This work underlined the need for oligosaccharides comprising multiple O-Ag repeats [25]. The synthetic feasibility of such larger SF3a oligosaccharides diverging by their endchain residue and O-acetylation pattern was demonstrated [26,27] and highly immunogenic TT-conjugates thereof were delivered (Hu et al., unpublished data). A bivalent SF2a/SF3a vaccine candidate was shown to be highly immunogenic in mice and no immune interference was detected [28]. Finally, well-defined frame-shifted oligosaccharides representing the *S. flexneri* 1b (SF1b) O-Ag were obtained (Le Guen et al., unpublished data) [29], on the way to promising SF1b glycoimmunogens. These remarkable advancements allowed the preclinical testing of sets of combinations of synthetic oligosaccharide-based conjugates representing the four *Shigella* strains to be targeted for the first licensed *Shigella* vaccine (International *Shigella* Vaccine meeting, Bill and Melinda Gates Foundation, Seattle, 2017). Available immunogenicity data support the concept (Phalipon et al., unpublished data). This constitutes a promising step towards the feasibility of this synthetic oligosaccharide-based strategy for developing a multivalent *Shigella* vaccine.

## 6. Conclusions

Despite numerous promising preclinical data [30], the fear of chemical synthesis—essentially owing to anticipated cost of goods and complexity—has somewhat impaired the development of the synthetic glycan-based vaccine strategy. It is noteworthy that Quimi-Hib^®^ [31] is so far the sole licensed vaccine of that type. However, this is without considering major recent developments in glycan chemical synthesis [30] and the increasingly acknowledged input of glycan chemoenzymatic synthesis [32], an alternative already explored successfully to obtain the SF2a 15-mer hapten [33]. With these groundbreaking and rapid technological advancements, the strategy represents more than ever a potentially cost-effective option for the design of high promise vaccines against major human infectious diseases. The demonstration of the proof of concept in humans for SF2a-TT15 assuredly contributed to the promotion of this approach. Obviously, the awaited findings of the ongoing phase II clinical studies, if supportive, will further strengthen its potential. A first-in-human evaluation of the quadrivalent formulation fulfilling all identified criteria will be the ultimate stage to assess the future of this original rationally designed synthetic oligosaccharide-based conjugate strategy for a broadly distributed *Shigella* vaccine.

## Data Availability

Not applicable.
